# Development of the IS-Score: A Novel Predictive Model for Shunt Requirement Following Aneurysmal Subarachnoid Hemorrhage

**DOI:** 10.1007/s12028-026-02461-y

**Published:** 2026-03-06

**Authors:** Lejla Islamagič, Ilies Djebbara, Troels Halfeld Nielsen, Sune Munthe

**Affiliations:** 1 Department of Neurosurgery, Odense University Hospital, Kløvervænget 47, Entrance 44, 5000 Odense, Denmark; 2 Department of Clinical Research, University of Southern Denmark, Odense, Denmark; 3 BRIDGE (Brain Research – Interdisciplinary Guided Excellence), University of Southern Denmark, Odense, Denmark

**Keywords:** Subarachnoid hemorrhage, Secondary hydrocephalus, Ventriculoperitoneal shunt, SAH-VP score, MAGE score

## Abstract

**Background:**

Chronic hydrocephalus after aneurysmal subarachnoid hemorrhage is a common condition that can require shunting of cerebrospinal fluid to the peritoneal cavity [ventriculoperitoneal shunting (VPS)]. Several predictive scoring systems have been developed to estimate the risk of requiring VPS, most recently the Mayo age, grade, EVD (MAGE) score and the SAH-VP score. The aim of this study was to identify predictors of ventriculoperitoneal shunt dependency and to develop a new predictive score to compare with the MAGE and SAH-VP scores.

**Methods:**

A retrospective single-center cohort study of 220 patients admitted with computed tomography (CT)-verified aneurysmal subarachnoid hemorrhage (aSAH), of whom 114 required extraventricular drainage, between 1 September 2018 and 21 January 2025, was conducted. They were categorized into two groups: those requiring VPS (*n* = 62) and those who did not (*n* = 52). Univariate and multivariate analyses (least absolute shrinkage and selection operator logistic regression combined with the extended Bayesian information criterion for the latter) were performed. Statistically significant variables were entered into a generalized linear model. A clinical risk score was developed by scaling and rounding the bootstrapped regression coefficients relative to the smallest significant effect.

**Results:**

Least absolute shrinkage and selection operator (LASSO) regression identified seven predictors: intensive care unit (ICU) stay ≥10 days (OR 10.85), World Federation of Neurological Surgeons (WFNS) score of 5 (OR 1.35), EVD duration≥14 days (OR 1.73), two or more failed weaning attempts (OR 5.38), clamp failure (OR 2.99), cerebrospinal fuid (CSF) output>200 mL in the frst 3 days (OR 3.67), and failed closure after weaning (OR 4.42). The final model, named the Islamagič Shunt score (IS-score) demonstrated a bootstrapped area under the receiver operating characteristic curve (AUC) of 0.914 (95% CI 0.87–0.95). In comparison, the SAH-VP yielded an AUC of 0.736 (95% CI 0.66–0.80) and the MAGE score an AUC of 0.791 (95% CI 0.73–0.85).

**Conclusions:**

The IS-score demonstrated superior predictive performance for VPS following aSAH compared with the MAGE and SAH-VP scores, but further external validation is needed.

## Introduction

Aneurysmal subarachnoid hemorrhage (aSAH) is an acute, life-threatening condition associated with high morbidity and mortality. A common complication is the development of hydrocephalus requiring extraventricular drainage (EVD) [1]. The occurrence of hydrocephalus is, on its own, a predictor for worse Glasgow Outcome Scale (GOS) category [1].

Chronic hydrocephalus is associated with a high likelihood of EVD treatment requiring ventriculoperitoneal shunting (VPS) [2, 3]. Risk factors associated with the development of chronic hydrocephalus and the need for VPS include age over 65 years [1, 4–8]; aneurysm located in the anterior communicating artery (ACOM) [1, 2, 7, 9], middle cerebral artery (MCA) [1, 10], posterior circulation [4, 11–13], or posterior communicating artery (PCOM) [1, 2, 14]; higher Hunt and Hess grade (HHG) [1, 3, 4, 6, 8, 12, 13, 15–18]; higher original or modified Fisher grade (mFG) at admission [1, 3, 4, 6, 8, 12–17]; higher World Federation of Neurological Surgeons (WFNS) score [6, 19]; intraventricular hemorrhage (IVH) [4, 6–8, 11, 13, 15–17, 20, 21]; and vasospasm [6, 13, 15, 18]. The risk of requiring VPS is also increased by prolonged EVD use [4, 19], daily drainage output > 200 mL on day 0–2 [22], clinical changes or radiographic hydrocephalus and corresponding wean failure [23], multiple failed wean/closure trials [19], and any wean/clamp trial failure [22].

*Ascanio et al.* found that after the second and third clamp trial, 60% and 38.9% of patients did not require VPS, respectively [24]. However, there remains a risk of delayed hydrocephalus after EVD removal. Antunes et al. found the prevalence to be 7.8% in patients passing the initial clamp trial versus 14.3% in those failing the initial trial and 80% in those failing multiple trials [25], prompting awareness even after a successful clamp trial.

No international guidelines currently exist regarding the discontinuation of EVD treatment. A Scandinavian neurosurgical survey on the management of EVD discontinuation in patients with hydrocephalus following aSAH showed 75% of responders based their discontinuation strategy on the patient’s clinical status (45% relied primarily on the Glasgow Coma Scale) and drainage volume [26]. There was agreement of initiating discontinuation at 4–7 days after ictus if the patient was stable, had a drainage output of < 150 mL/day, and intracranial pressure (ICP) < 15 mm Hg [26]. A US institutional survey showed that 78% of institutions preferred a gradual EVD weaning strategy with a continuously open EVD in patients with secured aneurysms [27]. Scoring systems such as the Mayo age, grade, EVD (MAGE) score [22], and predictive models have emerged over the years in attempts to determine the risk of VPS after aSAH. Pereira et al.[19] recently proposed a new scoring system, the SAH-VP score, which they claimed predicted the risk of VPS better than the MAGE score.

The present study aimed to externally validate the predictive accuracy of the MAGE and SAH-VP scoring systems in our patient cohort and to investigate whether we could create a novel scoring system to predict the risk of VPS more accurately.

## Methods

### Study design and inclusion criteria

This was a retrospective single-center cohort study of all patients admitted to the Department of Neurosurgery in the Region of Southern Denmark between 1 September 2018 and 21 January 2025 with a computed tomography (CT)-verified SAH due to spontaneous rupture of an intracerebral aneurysm. We excluded patients who did not receive neurosurgical or neuroradiological intervention due to severe SAH and low GCS. Approval to access and process patient data for research purposes was obtained from the Regional Council (Regionsrådet). All data were handled in compliance with data protection legislation, and no direct patient contact or intervention was involved.

Of the 220 patients initially identified, 151 (69.7%) required EVD. Of these, 37 patients who died during admission were excluded from the EVD analysis as they never received VPS and would have biased the study results. Consequently, 114 patients with EVD were included in the final analysis and were categorized as not requiring VPS (*n* = 52) or requiring VPS (*n* = 62). Of the 69 patients not requiring EVD, 2 patients later needed VPS due to chronic hydrocephalus found after transfer to a rehabilitation center, but they were excluded from our EVD group.

### Intervention

Patients underwent standard-care management, and all patients were initially admitted to the intensive care unit (ICU), whether they were intubated or not. Management included tight blood pressure monitoring (keeping systolic pressure < 160 mm Hg), strict bed rest, initiation of nimodipine, and external ventricular drain placement when indicated. Before treatment, the EVD resistance was set at 20 cmH20. After treatment, the EVD resistance was adjusted according to the clinical condition of the patient and the ICP.

Following digital subtraction angiography, the treatment strategy was determined collaboratively by neurosurgeons and neuroradiologists. Patients were either treated endovascularly (mostly by coiling, but some by stenting or both) or with open microsurgery in the form of a craniotomy with clipping of the aneurysm (mostly without coiling or stenting) within the first 24 h. Those with stenting alone or a combination of coiling, clipping, and stenting constituted such a small a part of the cohort that they were not statistically significant individually and are presented in Table 2 under ‘other’ aneurysm characteristics.

### Data collection and statistical analysis

We followed the Strengthening the Reporting of Observational Studies in Epidemiology (STROBE) guidelines for cohort studies. Data were collected (by LI and ID) from the electronic medical records on age, sex, comorbidity, smoking and alcohol, initial Glasgow Coma Scale (CGS), aSAH variables, HHG, modified Fisher grade (mFG), WFNS score, MAGE score [22], SAH-VP score, EVD treatment, length of ICU stay, and mortality.

To allow external validation using the study by Pereira et al. [19], we chose to analyze the same EVD parameters, i.e., mean drainage volume above or below 200 mL a day on days 0–2, two or more wean failures, any clamp failures, and age over 65 years. A gradual wean failure or rapid clamp failure were defined as not being able to increase the EVD drain resistance (to 25 cmH2O) or to rapidly clamp the drain to test whether the patient was drain-dependent due to increased ICP or clinical deterioration. The decision to gradually wean or rapidly clamp the EVD was a clinical decision made for the individual patient and only after the aneurysm was treated.

Failed closure after weaning was defined as being able to gradually increase the EVD resistance (by an increment of 5 cmH20) to 25 cmH2O but not being able to clamp the EVD, either due to increased ICP or clinical deterioration due to drain-dependency. EVD dysfunction was defined as an EVD not functioning normally due to either clotting or initial malplacement. All patients who were drain-dependent received VPS, but other forms of shunting could have been considered (such as atrial or pleural shunting).

The first 15 patients were reviewed jointly to ensure uniform reporting. Data were entered into Microsoft Excel® and analyzed using STATA 18 Inc^®^. Statistical significance was defined as a *p*-value < 0.05. Shapiro–Wilk test and skewness and kurtosis tests indicated that the data were nonnormally distributed; therefore, data are presented as median values with 95% confidence intervals (CI). Mann–Whitney *U *tests and chi-squared tests (or Fisher’s exact test if frequencies were < 5) were used to compare patient groups on sociodemographic and clinical variables.

For multivariate analysis, candidate predictors for VPS were selected from the univariate analyses and existing literature on factors that increase the risk of VPS (see results under ‘Multivariate analysis’ for further details). Least absolute shrinkage and selection operator (LASSO) logistic regression was applied using tenfold cross-validation to reduce model overfitting and optimize predictor selection. The extended Bayesian information criterion (EBIC) was used to choose the optimal shrinkage parameter (*λ*). No variables were forced into the model, and variables with non-zero coefficients were retained for multivariable modelling. Variables were entered into a generalized linear model (GLM) with a binomial distribution and a logit link function. Odds ratios (ORs), 95% CIs, and *p*-values were reported. Coefficients were internally validated using nonparametric bootstrapping (1000 replicates) with percentile-based CIs derived from the bootstrap distribution. Replicates with perfect prediction or convergence errors were excluded from the final estimates. Discrimination was assessed using the area under the receiver operating characteristic curve (AUC), which was bootstrapped using 1000 manually sampled replicates with AUCs collected and summarized from each iteration to ensure stability of the estimates. Model performance was compared with the SAH-VP and MAGE scoring systems. Calibration was assessed visually and via the Hosmer–Lemeshow goodness-of-fit test. Variance inflation factors (VIF) were used to rule out multicollinearity.

A clinical risk score was developed by scaling and rounding the bootstrapped regression coefficients relative to the smallest significant effect (WFNS score). This produced an integer-based scoring system, allowing for stratification into clinically meaningful risk categories.

## Results

### Demographic data and risk of VPS

Demographic data for all patients and for EVD patients either receiving or not receiving VPS are presented in Table 1. A total of 220 patients with aSAH were included, of which 114 required EVD placement (after exclusion of 37 patients with EVD who died during admission and therefore could not be evaluated for VPS placement).

**Table 1:** Sociodemographic and clinical variables for all patients with aneurysmal subarachnoid hemorrhage (aSAH) and for those treated with extraventricular drainage (EVD) categorized as either with or without ventriculoperitoneal shunting (VPS)

	Total patients with aSAH		Patients treated with EVD				*p*-Value
	(*n* = 220)	Percentage (%)	No VPS (*n* = 52)	Percentage (%)	VPS (*n* = 62)	Percentage (%)	
*Age* (years)							
Males, median (95% CI)	59 (55–63)		59 (51.8–66.2)		64 (57.5–70.5)		0.65
Female, median (95% CI)	61 (59–64)		58 (53.6–62.3)		61 (57.8–64.2)		0.15
*Sex*							
Male	62	28.2	11	21.2	18	29	0.21
Female	158	71.8	41	78.8	44	71	0.40
*Comorbidity and lifestyle factors*							
Hypertension	79	36.1	13	25	30	48.4	0.01
Hypercholesterolemia	24	11.1	4	7.69	7	11.3	0.52
Antiplatelet therapy	17	7.7	3	5.77	4	6.45	0.88
Anticoagulant therapy	4	1.8	1	1.92	2	3.23	0.67
Current smoker	69	31.4	15	28.8	16	25.8	0.71
Previous smoker	40	18.2	13	25	7	11.3	0.06
Never smoked	51	23.2	12	23.1	20	32.3	0.28
Unknown	59	26.8	12	23.1	19	30.6	0.37
Alcohol (< 10 units weekly)	30	13.6	6	11.5	5	8.06	0.53
Alcohol (≥ 10 units weekly)	60	27.3	13	25	13	20.9	0.61
No alcohol	44	20.0	3	5.77	0	0	0.89
Unknown	76	34.5	17	32.7	26	41.9	0.31
*Mortality*							
Died during hospital stay	41	18.6	0	0	0	0	-
Died within 1 year after admission	19	8.6	5	9.61	12	19.4	0.15
Died within 2 years after admission	2	1	0	0	1	1.61	0.36

The *p*-values represent the statistical significance of comparisons between the no VPS and VPS groups

Among all 220 patients, there were significantly more female than male patients (*p* < 0.05), with female patients accounting for approximately 72% of aSAH cases. Among the EVD group, there was no statistical association between VPS and sex (*p* = 0.3). No age differences were found between female and male patients in the overall group (*p* = 0.28), in the no VPS group (*p* = 0.44), or in the VPS group (*p* = 0.92).

Patients who were treated with VPS were significantly more likely to have hypertension (*p* = 0.01) and not have diabetes (*p* = 0.04) compared with the no VPS group. No statistical differences were found regarding smoking status or alcohol consumption between the no VPS and the VPS groups, or when comparing female and male patients in the overall group (*p* > 0.05).

Although 12 out of 19 deaths during the first year after admission were in the VPS group, this was not a significantly higher mortality compared with the no VPS group (*p* = 0.09).

### Clinical admission characteristics and risk of VPS

The occurrence of PCOM aneurysms was higher in patients not receiving VPS (*p* = 0.04), see Table 2. Receiving VPS was associated with an ICU stay of more than 10 days (*p* < 0.001) as well as mFG of 4, WFNS score of 5, and HHG of 4–5 (Table 2). Receiving VPS was also associated with MAGE scores of 2, 5, and 6 and SAH-VP scores of 7–10 (all *p* < 0.01) (Table 3).

**Table 2:** Clinical characteristics, treatment, and complications for all patients with aneurysmal subarachnoid hemorrhage (aSAH) and for those treated with extraventricular drainage (EVD) categorized as either with or without ventriculoperitoneal shunting (VPS)

	Total patients with aSAH		Patients treated with EVD				*p*-Value
	(*n* = 220)	Percentage (%)	No VPS (*n* = 52)	Percentage (%)	VPS (*n* = 62)	Percentage (%)	
*Initial GCS*							
15	75	34.1	18	34.6	10	16.1	0.13
13–14	63	28.6	21	40.4	17	27.4	0.52
9–12	13	5.9	7	13.5	3	4.84	0.26
6–8	19	8.6	1	1.92	9	14.5	0.01
3–5	50	22.7	5	9.61	23	37.1	0.00
*Variables related to aneurysm and aSAH*							
1* aneurysm	180	81.8	41	78.8	53	85.5	0.22
2 or more aneurysms	40	18.2	11	21.2	9	14.5	0.65
Number of treated aneurysms (1 versus 2)	213/7	96.8/3.18	50/2	96.2/3.8	60/2	96.7/3.3	0.41
Size < 1 cm (largest diameter)	169	68.2	40	76.9	47	75.8	0.25
Size ≥ 1 cm (largest diameter)	79	31.9	12	23.1	15	24.2	0.65
ACOM	90	40.9	20	38.5	31	50	0.22
PCOM	25	11.4	7	13.5	2	3.22	0.04
ACA	2	0.90	1	1.92	0	0	0.27
MCA	42	19.1	6	11.5	11	17.7	0.35
ICA	21	9.55	11	21.2	9	14.5	0.35
AChorA	1	0.45	0	0	0	0	–
PICA	11	5	4	7.69	5	8.09	0.94
PCLA	9	4.09	3	5.77	0	0	0.06
SCA	2	0.91	2	3.85	1	1.61	0.46
VA	3	1.36	0	8	3	4.85	0.11
BA	18	8.18	4	7.69	4	6.45	0.80
Intracranial hematoma in relation to aneurysm	68	30.2	13	25	21	33.9	0.30
Rebleeding during hospitalization	39	17.9	6	11.5	14	22.6	0.12
Complication to endovascular procedure	17	8.3	2	3.85	4	6.45	0.54
Seizures at debut	35	16.1	6	11.5	14	22.6	0.12
Seizures during hospitalization	10	4.6	4	7.69	4	6.45	0.80
Coiling	158	71.8	38	73.1	48	77.4	0.28
Clipping	50	22.7	11	21.2	8	12.9	0.49
Other	12	5.45	3	5.77	6	9.67	0.32
Clinical vasospasm	58	28.6	15	28.8	15	24.2	0.57
Radiological vasospasm	58	28.6	16	30.8	16	25.8	0.56
Transcranial Doppler vasospasm	26	14.8	8	15.4	6	9.67	0.36
*Length of ICU stay*							
< 10 days	90	40	16	30.8	3	4.85	0.00**
≥ 10 days	130	60	36	69.2	59	95.15	0.00***
*Grading systems*							
mFG							
1	16	7.3	4	7.69	2	3.22	0.41
2	13	5.9	2	3.85	5	8.06	0.26
3	83	37.7	23	44.2	13	20.9	0.10
4	108	47.1	23	44.2	42	67.7	0.02
WFNS							
1	76	34.6	17	32.7	11	17.7	0.26
2	52	23.6	18	34.6	14	22.6	0.48
3	10	4.6	5	9.62	1	1.61	0.10
4	26	11.8	7	13.5	9	14.5	0.62
5	56	25.5	5	9.62	27	43.5	0.00
HHG							
1	35	15.9	6	11.5	7	11.3	0.78
2	81	36.8	27	51.9	14	22.6	0.04
3	26	11.8	11	21.2	8	12.9	0.49
4	23	10.5	1	1.92	9	14.5	0.01
5	55	25	7	13.5	24	38.7	0.00

*ACOM* anterior communicating artery, *PCOM* posterior communicating artery, *ACA* anterior cerebral artery, *MCA* medial cerebral artery, *ICA* internal carotid artery, *AChorA* anterior choroidal artery, *PICA* posterior inferior cerebellar artery, *PCLA* pericallosal artery, *SCA* superior cerebellar artery, *BA* basilar artery, *VA* vertebral artery

The *p*-values represent the statistical significance of comparisons between the no VPS and VPS groups

*The number of aneurysms is higher than the number of patients as some patients had multiple unruptured aneurysms

**Significantly lower risk of VPS

***Significantly higher risk of VPS

**Table 3:** MAGE scores and SAH-VP scores for risk of ventriculoperitoneal shunting (VPS) in patients treated with extraventricular drainage (EVD), stratified by receiving or not receiving VPS

	Patients treated with EVD						
	No VPS (*n* = 52)	Percentage (%)		VPS (*n* = 62)	Percentage (%)		*p*-Value
*MAGE score*							
0	16	30.8		3	4.8		0.004
1	22		42.3	22		35.5	0.14
2	10		19.2	14		22.6	0.06
3	3		5.8	3		4.8	0.31
4	1		1.9	2		3.2	0.58
5	0		0	0		0	0
6	0		0	3		4.8	0.004
*SAH-VP score*							
0–2	49		94.2	47		75.8	0.60
3–4	2		3.8	4		6.5	0.08
5–6	1		1.9	5		8.1	0.01
7–10	0		0	6		9.7	< 0.01

The *p*-values represent the statistical significance of comparisons between the no VPS and VPS groups

Chi-squared distribution (gamma distribution) showed an increased risk of VPS with poorer GCS (*p* < 0.01). Binomial testing found the shift primarily occurred for patients in the two lowest groups, i.e., GCS < 8. Gamma distribution also found an increased risk of VPS with worse mFG (*p* = 0.025) and MAGE score (*p* < 0.01).

### EVD treatment and risk of VPS

Univariate analyses of variables related to EVD treatment are presented in Table 4. Variables associated with an increased risk of VPS after EVD treatment were EVD placed the same day (*p* < 0.01), daily output volume > 200 mL (*p* = 0.04), two or more failed wean trials (*p* < 0.01), two or more failed closures after weaning (*p* < 0.01), any clamp failure (*p* < 0.01), and > 14 days with an open EVD (*p* < 0.01).

**Table 4:** EVD-related parameters in patients treated with extraventricular drainage (EVD) and categorized as either with or without ventriculoperitoneal shunting (VPS)

	All patients with EVD (*n* = 114)	Percentage (%)	No VPS (*n* = 52)	Percentage (%)	VPS (*n* = 62)	Percentage (%)	*p*-Value
*EVD treatment*							
Placed same day as admission	87	76.3	39	75	50	80.6	0.00
Placed after first day of admission	27	23.7	13	25	12	19.4	0.13
Daily output (days 0–2)							
< 200 mL	65	57.0	35	67.3	31	50	0.008
≥ 200 mL	49	42.9	17	32.7	31	50	< 0.01
Failed weaning (1 try)	19	16.6	8	15.4	8	12.9	0.70
Failed weaning (+ 2 tries)	30	26.3	5	9.61	24	38.7	< 0.01
Failed closure after weaning (1 try)	16	14.0	4	7.69	9	14.5	0.52
Failed closure after weaning (+ 2 tries)	21	18.4	3	5.77	10	16.1	< 0.01
Failed rapid closure (1 try)	14	12.3	4	7.69	9	14.5	0.25
Failed rapid closure (+ 2 tries)	14	12.3	3	5.77	10	16.1	0.08
Any clamp failure	60	52.6	15	28.8	38	61.3	< 0.01
EVD dysfunction	40	35.1	9	17.3	16	25.8	0.28
EVD-related infection	7	6.14	3	5.77	4	6.45	0.88
Days with an open EVD (> 14 days)	70	61.4	21	40.4	44	70.9	< 0.01

The *p*-values represent the statistical significance of comparisons between the no VPS and VPS groups

### Multivariate analysis

**Fig. 1:**
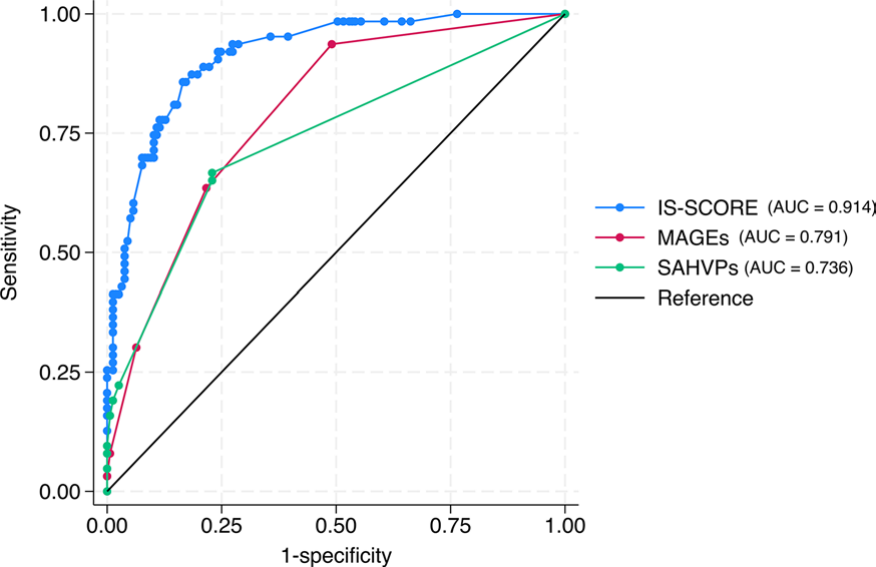
ROC curves for the IS-, SAH-VP, and MAGE scores. ROC curves showing greater predictive performance of the IS-score (AUC 0.914) compared with the MAGE (AUC 0.791) and SAH-VP (AUC 0.736) scores for ventriculoperitoneal shunt dependency after aneurysmal subarachnoid hemorrhage

LASSO regression identified seven predictors with non-zero coefficients: ICU stay ≥ 10 days, WFNS score of 5, EVD duration ≥ 14 days, two or more failed weaning attempts, clamp failure, cerebrospinal fluid (CSF) output > 200 mL in the first 3 days, and failed closure after weaning (Table 5). There was no evidence of multicollinearity (all VIFs < 2). Model calibration was acceptable (Hosmer–Lemeshow *p* > 0.05). The final model demonstrated excellent discrimination, with a bootstrapped AUC of 0.914 (95% CI 0.87–0.95). In comparison, the SAH-VP score yielded an AUC of 0.736 (95% CI 0.66–0.80), and the MAGE score yielded an AUC of 0.791 (95% CI 0.73–0.85) (see Fig. 1).

**Table 5:** The seven variables significantly associated with risk of requiring ventriculoperitoneal shunting (VPS) after initiation of extraventricular drainage (EVD), showing the number of weighted points for each variable

Predictor	Bootstrapped odds ratio (95% CI)	*p*-Value	Points
ICU stay ≥ 10 days	10.85 (2.4–48.3)	0.002	8
WFNS score = 5	1.35 (1.00–1.82)	0.049	6
Failed weaning (≥ 2 attempts)	5.38 (1.25–23.3)	0.024	5
Clamp failure	2.99 (0.92–9.69)	0.068	4
CSF output ≥ 200 mL (day 0–2)	3.67 (1.37–9.85)	0.010	4
Failed closure after weaning	4.42 (1.09–17.9)	0.037	2
EVD duration ≥ 14 days	1.73 (0.53–5.65)	0.364*	1

The total score ranged from 0 to 30 points. On the basis of the total score, patients were stratified into three risk categories: low risk (0–9 points): 5.7% VPS incidence, intermediate risk (10–17 points): 28.6% VPS incidence, and high risk (≥ 18 points): 67.2% VPS incidence

*Nonsignificant but chosen to be included in the model through LASSO regression

### Development of a new clinical risk score

In the GLM, an EVD duration ≥ 14 days was not significant (*p* = 0.36). Nevertheless, it was kept in the risk score as it was selected by the LASSO regression, which emphasizes predictive performance over statistical interference and thus selects variables that contribute to the overall model discrimination despite the statistical significance. EVD duration ≥ 14 days was included in the risk score to reflect its potential predictive contribution. Bootstrap regression coefficients were used to assign weighted points to each of the seven variables (Table 5), creating a new scoring system we call the Islamagič Shunt score (IS-score). The total score ranged from 0 to 30 points and showed excellent discrimination (AUC 0.90). On the basis of the total score, patients were stratified into three risk categories:

- Low risk (0–9 points): 5.7% VPS incidence
- Intermediate risk (10–17 points): 28.6% VPS incidence
- High risk (≥ 18 points): 67.2% VPS incidence

## Discussion

This study investigated risk factors associated with receiving ventriculoperitoneal shunting after aneurysmal subarachnoid hemorrhage, alongside testing the clinical accuracy of the MAGE score and the recently introduced SAH-VP score in predicting a patient’s need for VPS. The results from our patient cohort showed the SAH-VP score to be less accurate at predicting the need for VPS compared with the MAGE score, and both scores were outperformed by our newly proposed IS-score. The IS-score is not intended for early prediction but rather for stratification after the acute phase of aSAH, once relevant time-dependent parameters are available.

These results are contrary to the findings of Pereira et al. [19], who found their SAH-VP model better predicted the risk of VPS compared with the MAGE score. The SAH-VP score was not significant on multivariate analysis unlike the MAGE score. Although higher SAH-VP scores were associated with receiving VPS (*p* = 0.046, data not shown in tables), the distribution of SAH-VP scores was highly uneven, with approximately 82% of patients being in group 1. This greatly reduced the statistical power and reliability when interpreting the association with VPS and increased the risk of type-I errors (false positives). A major confounding factor in the study by Periera et al. [19] was high incidence of EVD-related infections (42.8%), creating bias in their scoring system and making our cohort less comparable [only 7 out of 114 patients (6.1%) treated with EVD in our cohort had ventriculitis]. Conversely, they did not find the mean daily EVD output the first 3 days after placement to be a significant predictive factor for VPS in either uni- or multivariate analysis. This suggests the need for further validation studies of the SAH-VP scoring system across larger and more diverse populations to investigate whether it can accurately stratify VPS risk after aSAH.

Our univariate analyses confirmed the significance of EVD management in determining VPS risk. Specifically, daily output exceeding 200 mL and failures in weaning trials appeared to be critical in predicting VPS, indicating that a proactive approach in EVD management—especially monitoring output volumes and promptly addressing weaning failures—could mitigate the risk of chronic complications.

The inverse relationship between ICU stay duration and VPS risk raises important considerations for clinical decision-making. We included ICU stay > 10 days as a variable to assess whether prolonged critical illness itself—independent of any specific identifiable complication—was associated with the subsequent need for ventriculoperitoneal shunt (VPS) placement. Its substantial contribution to the predictive model suggests that extended ICU dependency reflects a composite signal of neurological injury, impaired CSF circulation recovery, and overall disease severity, all of which are established risk factors for shunt-dependent hydrocephalus following subarachnoid hemorrhage. Thus, ICU length of stay should be interpreted not as a causal factor for VPS requirement, but as a clinically meaningful surrogate marker of sustained physiological instability and delayed neurological recovery, which together identify a subgroup of patients at higher risk of developing persistent CSF circulation failure.

Parameters such as the HHG and the WFNS demonstrated a strong association with VPS, particularly at the extremes of their scoring ranges. The finding that HHG of 2 was disproportionately more common in the non-VPS group may reflect an atypical manifestation of clinical severity that correlates with a lesser risk of VPS. This needs to be verified in other studies. The significant correlation of lower GCS scores with increased VPS risk underscores the severity of aSAH and is also a factor that affects the prognostic outcome on its own.

Two time-dependent variables were included in our model: an ICU stay of more than 10 days and an EVD duration of more than 14 days. The length of ICU stay was the variable with the highest number of weighted points (8 points), while EVD duration of more than 14 days had the lowest number (1 point). Decisions regarding EVD weaning or clamping were individualized and based on the patient’s neurological status, intracranial pressure, and recent CSF drainage volumes, rather than by any predetermined duration of EVD placement. This decision depends on multiple factors, such as the patient’s overall clinical status, whether the aneurysm has been sufficiently treated, and the intracranial pressure trends. Patients who have a low drainage need and whose EVD treatment can be quickly discontinued have a very low risk of requiring VPS and would score low in our model prediction. Therefore, these two time-dependent variables do not affect the model’s early clinical usage, but they strengthen the clinical indication for VPS with prolonged ICU stay or EVD use.

The findings from this study underscore the importance of integrated management strategies in patients with aSAH, particularly concerning EVD treatment and VPS risk assessment. The IS-score provides a practical tool for identification of patients likely to require VPS, potentially guiding monitoring intensity, resource allocation, and discharge planning. The interplay between demographic factors, GCS, and scoring systems highlights the complexity of managing patients with aSAH and presents opportunities for the development of tailored protocols aimed at reducing morbidity. It is also important to state that the model determines the requirement for VPS following aSAH, which can be achieved by not only ventriculoperitoneal shunting, but also ventriculoatrial or ventriculopleural shunting.

Future studies are essential to further investigate the predictive ability of the IS, MAGE, and SAH-VP scoring systems, alongside the refinement of clinical guidelines to optimize the outcomes for patients with aneurysmal subarachnoid hemorrhage.

### Limitations

A limitation of our study is the single-center design and the relatively small number of patients included. There is a potential for practice bias, and we have not investigated the role of ethnicity. There is a risk of information bias as two of the authors extracted and reviewed the data, but this was minimized by charting the first 15 patients jointly. Despite the current internal validation, the IS-score remains to be externally validated to assess its generalizability across different clinical settings. Ideally, future studies should include a sufficiently large and diverse patient population to allow for robust statistical evaluation of predictive accuracy. A multicenter Danish or Nordic cohort would be particularly valuable, as it would provide a larger sample size and more heterogeneous data, while benefiting from relatively uniform management protocols, including standardized EVD practices. Such an approach would enable a comprehensive assessment of the IS-score’s performance and facilitate direct comparison with existing predictive models such as the MAGE and SAH-VP scores.

In our cohort, EVD management was not standardized regarding drainage height or systematic weaning after aneurysm occlusion. Instead, EVD resistance (i.e., drainage level in cmH2O) was dynamically adjusted by the treating clinicians according to the patient’s neurological status and intracranial pressure (ICP). The threshold of > 200 ml/day should be understood as an operational marker of high CSF dependency under real-world, clinician-guided EVD management rather than as a physiologically absolute cutoff.

The inclusion of time-dependent variables, such as ICU stay and EVD duration, limits the use of the IS score for early prediction of shunt dependency. Instead, the score is intended to aid in stratifying patients after the acute phase of aSAH, when these parameters are known and clinical management decisions regarding permanent CSF diversion are being considered. Another limitation is that intraventricular hemorrhage (IVH) was not analyzed as an independent variable, as we used the modified Fisher grade, which already incorporates the presence and amount of IVH. Consequently, the specific contribution of IVH to shunt dependency could not be separately evaluated.

## Conclusions

The IS-score demonstrated superior predictive performance for ventriculoperitoneal shunting following aneurysmal subarachnoid hemorrhage compared with the MAGE and SAH-VP scoring systems.
