# Correction: Development of the IS-Score: A Novel Predictive Model for Shunt Requirement Following Aneurysmal Subarachnoid Hemorrhage

**DOI:** 10.1007/s12028-026-02537-9

**Published:** 2026-06-15

**Authors:** Lejla Islamagič, Ilies Djebbara, Troels Halfeld Nielsen, Sune Munthe

**Affiliations:** 1 Department of Neurosurgery, Odense University Hospital, Kløvervænget 47, Entrance 44, 5000 Odense, Denmark; 2 Department of Clinical Research, University of Southern Denmark, Odense, Denmark; 3 BRIDGE (Brain Research – Interdisciplinary Guided Excellence), University of Southern Denmark, Odense, Denmark

**Correction to: Neurocritical Care** 10.1007/s12028-026-02461-y.

The original article has been corrected to update the WFNS score point in the following places:

In the abstract, the WFNS score should be specified as a score of 5.

Table 5: WFNS = 5 (not per point)

Results, Multivariate analysis: “LASSO regression identified seven predictors with non-zero coefficients: ICU stay ≥ 10 days, WFNS score of 5,…''

Some of the values in the Results section were inadvertently switched. This is now corrected to read:

Results: Least absolute shrinkage and selection operator (LASSO) regression identifed seven predictors: intensive care unit (ICU) stay ≥10 days (OR 10.85), World Federation of Neurological Surgeons (WFNS) score of 5 (OR 1.35), EVD duration ≥14 days (OR 1.73), two or more failed weaning attempts (OR 5.38), clamp failure (OR 2.99), cerebrospinal fuid (CSF) output >200 mL in the frst 3 days (OR 3.67), and failed closure after weaning (OR 4.42). The final model, named the Islamagič Shunt score (IS-score) demonstrated a bootstrapped area under the receiver operating characteristic curve (AUC) of 0.914 (95% CI 0.87–0.95). In comparison, the SAH-VP yielded an AUC of 0.736 (95% CI 0.66–0.80) and the MAGE score an AUC of 0.791 (95% CI 0.73–0.85).

instead of “Results: Least absolute shrinkage and selection operator (LASSO) regression identifed seven predictors: intensive care unit (ICU) stay ≥10 days (p<0.01), World Federation of Neurological Surgeons (WFNS) score (OR10.85), EVD duration ≥14 days (OR1.35), two or more failed weaning attempts (OR5.38), clamp failure (OR2.99), cerebrospinal fuid (CSF) output >200 mL in the frst 3 days (OR3.67), and failed closure after weaning (OR4.42). The final model, named the Islamagič Shunt score (IS-score) demonstrated a bootstrapped area under the receiver operating characteristic curve (AUC) of 0.914 (95% CI 0.87–0.95). In comparison, the SAH-VP yielded an AUC of 0.736 (95% CI 0.66–0.80) and the MAGE score an AUC of 0.791 (95% CI 0.73–0.85).”

